# Dregs of *Cardamine hupingshanensis* as a feed additive to improve the egg quality

**DOI:** 10.3389/fnut.2022.915865

**Published:** 2022-07-28

**Authors:** Feike Yu, Xiaohan Yu, Rongchen Liu, Dawei Guo, Qian Deng, Bingbing Liang, Xiaoye Liu, Hong Dong

**Affiliations:** ^1^Beijing Key Laboratory of Traditional Chinese Veterinary Medicine, Beijing University of Agriculture, Beijing, China; ^2^Beijing Traditional Chinese Veterinary Engineering Center, Beijing University of Agriculture, Beijing, China

**Keywords:** dregs of *Cardamine hupingshanensis*, feed additive, laying hens, production performance, egg equality

## Abstract

Natural plant herbs have many active compounds to prevent poultry diseases and improve poultry products. However, most herbs are supplied for human medicine. Thus, for economic and sustainable development purposes, the dregs of *Cardamine hupingshanensis* (DCH) were developed as a feed additive to improve the egg quality of laying hens in this work. Results showed that the contents of selenium in hen serum and eggs were increased under DCH feeding. Subsequently, DCH also promotes the antioxidant capacity and immunity of laying hens through the increase of superoxide dismutase (SOD), catalase (CAT), and immunoglobulin G (IgG) by ELISA detection. Finally, production performance and egg quality were further graded by monitoring the product condition and scoring the indexes of egg quality, which also displayed that DCH as a feed additive significantly improved the egg quality by enhancing yolk color, eggshell thickness, and egg shape index.

## Introduction

Natural herbal plants used as feed additives have extensive activities, including antioxidant, immune enhancement, anti-inflammation, etc (1, 2). On account of these activities, natural plants have been widely used as feed additives to improve poultry growth and reproductive performance (3, 4). In China, a new natural plant named *Cardamine hupingshanensis* reported by Bai et al. in 2008 had proved the potential value for its characteristic of selenium hyperaccumulation (5). *Cardamine hupingshanensis*, which rich selenium area promoted large growth and located in Hunan Province has an excellent ability to accumulate the environmental selenium (6). More than 70% of total selenium distributed in *C. hupingshanensis* was accumulated in the form of selenocysteine that participates in the formation of antioxidant enzymes in humans and animals (6, 7). In addition, recent studies also showed that selenite of the environment might be converted to selenate and finally assimilate this selenate into SeCys in the root tissue of *C. hupingshanensis via* the continuous action of ATP sulfurylase and APS reductase (8). Most recent reports revealed that the mechanisms of selenium tolerance in *C. hupingshanensis* were vacuolar storage, transamination, selenation, and the degradation of selenoproteins (8). In fact, *C. hupingshanensis* contains a wide range of antimicrobial active substances, such as polysaccharides, flavonoids, and so on (9, 10). However, most efforts on *C. hupingshanensis* research remain on the capacity of selenium hyperaccumulate, less advances put forward on the development of *C. hupingshanensis* in poultry feeding. Therefore, the aim of this work is to evaluate whether the dregs of *C. hupingshanensis* (DCH) can use as a feed additive to improve the egg quality of laying hens.

Scientific research shows that selenium is a cofactor of antioxidant enzymes in the form of SeCys (11). The deficiency of selenium will induce exudative diathesis, nutritional muscular dystrophy, and nutritional pancreatic atrophy in chicks (12–15). In addition, selenium benefits the protection against oxidative stress and improvement of various physiological processes including immunity enhancement, especially in the situation of selenium deficiency (16–19). However, hazardous effects on health were observed during the excess selenium intake, which was more than 200 μg per day for a substantial period of time, leading to selenosis, alopecia, type 2 diabetes, and so on (20–22). For chicken, excessive dietary selenium resulted in oxidative damage, immunity decrease, and a series of pathology changes (23). Research has shown that adding appropriate selenium to poultry feed can effectively enhance antioxidant capacity, improve growth performance and increase production efficiency (11, 24, 25). Nowadays in China, *C. hupingshanensis* is used for selenoprotein extraction and selenium supplement for human daily needs. However, the *C. hupingshanensis* will be produced many dregs during the industrial process of selenoprotein extraction, resulting in the waste of resources (7). Herein, we fed laying hens with DCH for 30 days in this study, and then evaluated the changes in selenium, antioxidant capacity, immunity, production performance, and egg laying to determine whether DCH can increase the selenium of laying hens and improve the egg quality.

## Materials and methods

### Source of DCH

*C. hupingshanensis* was produced by Huzhou Huerjin Biotechnology (Zhejiang, China), with a selenium concentration of 700 mg/kg. The DCH was the herbal dreg precipitation of selenoprotein extraction from *C. hupingshanensis* using ethanol, pectinase, or amylase by previously published methods with slight modifications (26).

### Animals and treatments

**Figure 1:**
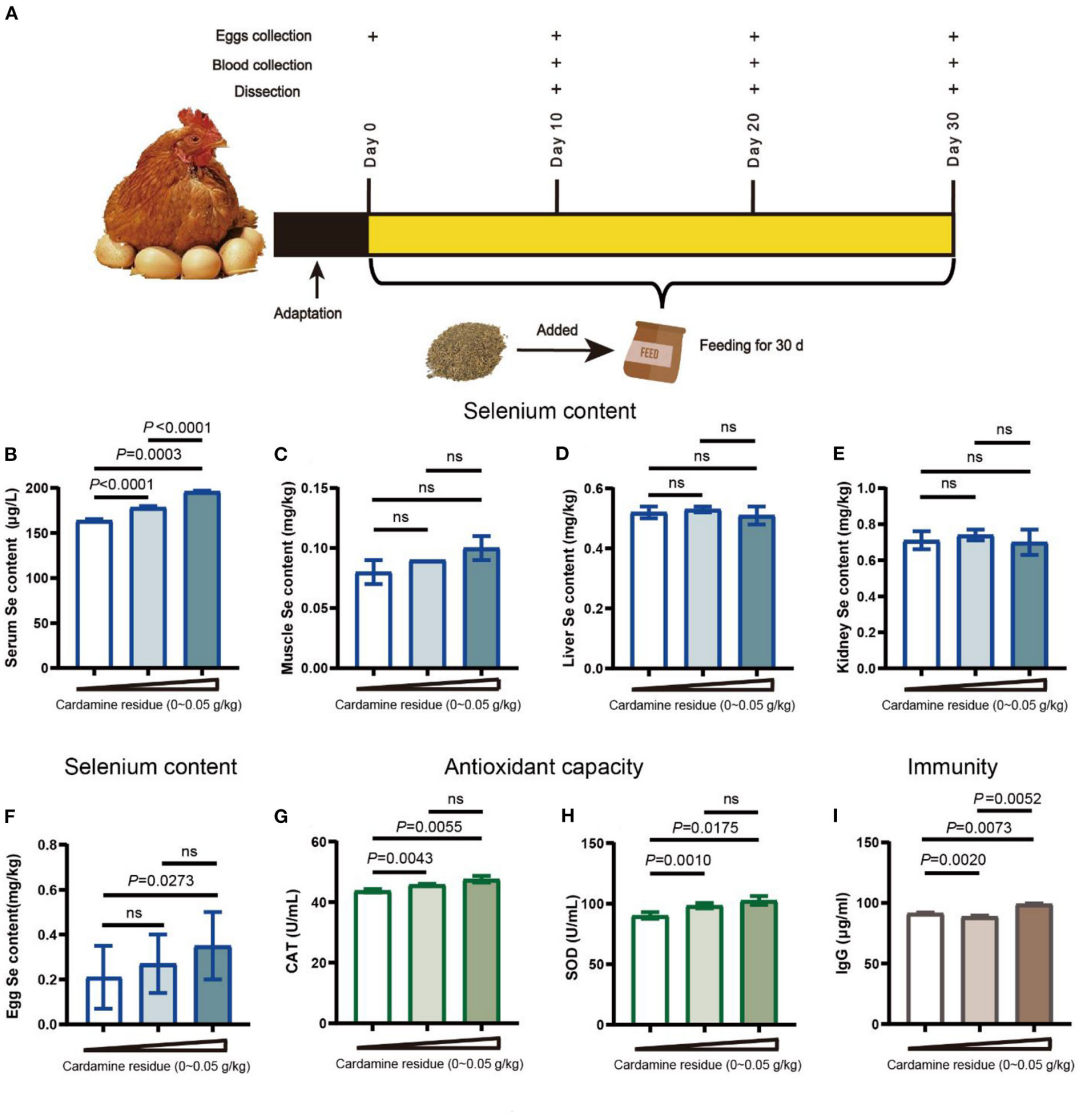
Effects of DCH on antioxidant capacity and immunity. **(A)** Workflow of the experiment. The laying hens were fed for 30 days and collected eggs on days 0, 10, 20, and 30 for production performance and eggs quality evaluation. Serum samples and organ samples were collected on the day 30 for growth performance evaluation. **(B–I)** Effects of DCH on serum selenium, organs selenium, egg selenium, antioxidant enzyme levels, and IgG level. **(B)** Selenium in serum of each group. **(C)** Selenium in muscle of each group. **(D)** Selenium in liver of each group. **(E)** Selenium in kidney of each group. **(F)** Selenium in eggs of each group. **(G,H)** The levels of antioxidative enzymes of SOD and CAT in serum of laying hens in each group. **(I)** IgG level in serum of laying hens in each group.

This study was carried out in Beijing Youth Farm (Pinggu, Beijing, China). One hundred and forty-four HY-LINE VARIETY BROWN laying hens were obtained at the 190-day old Beijing Youth Farm. These hens had similar egg production (Supplementary Tables 3, 4) and the immune program, including avian influenza, newcastle disease, and infectious bronchitis. After 2-week adaptation period, the laying hens were randomly divided into three groups, including the control group, low-dose, and high-dose DCH groups. Each group contained 16 laying hens and the experiments were repeated at least three times. The control group was only fed with basic diet, at the same time, the low-dose and high-dose DCH groups were fed a diet supplemented with 0.01 (kg of the feed) and 0.05 g/kg (kg of the feed) DCH, which containing 0.007 and 0.035 mg/kg selenium, respectively. The experiment period was 30 days, as well as the health status and production of laying hens were detected every day. The group information was detailed in Supplementary Table 1. The eggs and serum samples from hens were collected every 10 days for evaluation of product performance or antioxidant activity, and IgG level detention, respectively (Figure 1A and Supplementary Figure 1). The composition and nutrient levels of the basic diet were followed in Supplementary Table 2.

Hens were raised in three-tier cages and four hens were allotted to one cage (40^*^40^*^35 cm) on 15-h of light every day at room temperature. During the trial, all hens were fed 4 times daily and had free access to diet and water. Egg number and egg weight were recorded every day, and the status of the hens was observed and monitored daily.

### Sample collection

Two hens in each replicate were selected randomly for blood sample collection each 10 days. Blood samples were collected from the wing vein by vacuum tube, and then were marked and settled at room temperature for 1 h, followed by centrifugation at 3,000 r/min for 10 min. After separation and precipitation, the serum was separated into 1.5 ml centrifuge tubes and stored at −80 °C. After serum sampling from the wing vein, the hens were euthanized with pentobarbital sodium and dissected under aseptic conditions, and stored at −80 °C for the assessment of selenium content. Sixteen eggs in each repeat were randomly selected every 10 days and stored at 4 °C for detecting selenium contents, SOD level, and the assessment of egg quality.

The SOD (MM-33269O1), CAT (MM-33284O1), and IgG (MM-0505O2) levels of hens or eggs were measured by enzyme-linked immunosorbent assay (Jiangsu Meimian industrial, Jiangsu, China) according to the manufacturer's instructions.

### Se contents

According to the feed selenium determination method (GB/T 13883–2008) published by the Standardization Administration of China, selenium content in the diet was analyzed using hydride-atomic fluorescence spectrometry (AFS-9750, Created High-guarantee instrumentation, Beijing, China). On the basis of the China National Standard (GB 5009.93-2017), selenium contents of organs and eggs were analyzed by hydride-atomic fluorescence spectrometry (AFS-9750, Created High-guarantee instrumentation, Beijing, China). The serum selenium content was analyzed by graphite furnace atomic absorption spectrometry (27).

### Laying performance and egg quality

The production of hens in each group was recorded. In addition, the growth rate of egg production, the abnormal egg rate the growth rate of egg weight, and the ratio of feed and eggs were calculated throughout the experiment, according to the following formula:


$$\begin{array}{lll} Egg\;production\;rate & = & \frac{Total\;number\;of\;eggs}{Total\;number\;of\;hens}\times 100\%\\ Average\;egg\;weight & = & \frac{Egg\;weight}{Number\;of\;eggs}\times 100\%\\ Abnormal\;egg\;rate & = & \frac{Total\;number\;of\;abnormal\;eggs}{Total\;number\;of\;eggs}\times 100\% \end{array}$$

$$\begin{array}{lll} Growth\;rate\;of\;egg\;production & = & \frac{Egg\;production\;rate\;of\;30th\;day-Egg\;production\;rate\;of\;0th\;day}{Egg\;production\;rate\;of\;0th\;day}\times 100\%\\ Growth\;rate\;of\;average\;egg\;weight & = & \frac{Average\;egg\;weight\;of\;30th\;day-Average\;egg\;weight\;of\;0th\;day}{Average\;egg\;weight\;of\;0th\;day}\times 100\% \end{array}$$

$$\begin{array}{lll} Feed\;to\;egg\;ratio & = & \frac{Feed\;intake}{Egg\;weight}\times 100\%\\ Egg\;yolk\;ratio & = & \frac{Egg\;yolk\;weight}{Egg\;weight}\times 100\%\\ Egg\;shell\;ratio & = & \frac{Egg\;shell\;weight}{Egg\;weight}\times 100\% \end{array}$$

The egg shape index recorded the ratio of longitudinal diameter and transverse diameter of eggs, measured using the vernier caliper (Mitutoyo, Tokyo, Japan). Egg yolk color was measured using the Roche color fan (Robotmation, Tokyo, Japan). The egg yolk ratio indicated the proportion of egg yolk weight without egg shell and egg white to the weight of the whole egg. The eggshell thickness was the average of the thickness of the blunt end, middle part, and the sharp end of the egg, measured with a spiral micrometer (Weidu Electronics, Zhejiang, China). Eggshell ratio was the percentage of the weight of eggshell and whole egg. The chart of egg quality scoring standards was formulated according to the quality of eggs and prepared for subsequent analysis (Table 1). All of the equipment was from the Beijing Key Laboratory of Traditional Chinese Veterinary Medicine.

**Table 1:** Egg quality scoring standard.

**Score**	**Egg shape index** ^a^	**Yolk color** ^b^	**Yolk Ratio (%)** ^c^	**Eggshell thickness (mm)** ^d^	**Eggshell ratio (%)** ^e^
5	1.30 ≤ x ≤ 1.35	13–15	x≥30	≥0.4	x≥11
4	1.25 ≤ x <1.30;1.35 < x ≤ 1.40	10–12	25 ≤ x <30	0.35 ≤ x <0.40	10 ≤ x <11
3	1.20 ≤ x <1.25;1.40 < x ≤ 1.45	7–9	20 ≤ x <25	0.30 ≤ x <0.35	9 ≤ x <10
2	1.15 ≤ x <1.20;1.45 < x ≤ 1.50	4–6	15 ≤ x <20	0.25 ≤ x <0.30	8 ≤ x <9
1	1.10 ≤ x <1.20;1.50 < x ≤ 1.55	1–3	10 ≤ x <15	0.20 ≤ x <0.30	7 ≤ x <8
0	x <1.10;x>1.55	<1	x <10	x <0.2	x <7

*^a^Egg shape index, calculated as above between 1.3–1.53 was regarded as standard egg shape. ^b^Egg yolk color was judged by Roche yolk color fan, which is divided into 15 level, No. 15 represents the highest nutritional value. ^c^Egg quality increases with Egg yolk ratio, Eggs with a yolk ratio of more than 30% are considered superior eggs. ^d^Eggshell thickness is positively correlated with eggshell quality and it was regarded as superior egg when eggshell thickness exceeds 0.35 mm, there was 85% chance of smash if the thickness of eggshell was <0.25 mm. ^e^Eggshell ratio higher than 11% is considered superior egg, and lower than 7% is seen as inferior egg*.

### Statistical analysis

All data were shown as means ± standard deviation and analyzed using GraphPad Prism 8 (GraphPad Software, USA). Significant differences among groups were conducted using *t*-test by one-way analysis of variance (ANOVA). The results were assumed to be significant when *p* < 0.05.

## Results

### DCH increases selenium in hen serum and egg

Plant *C. hupingshanensis* has the ability to accumulate the selenium from the growth soil (6). To make full use of the selenium in this plant, we used the dregs of *C. hupingshanensis* (DCH) as an additive feeding the hens for 30 days and investigated whether DCH raises the selenium of hens *in vivo*. As expected, the serum selenium was significant increases in both low-dose (178.37 μg/L) and high-dose groups (196.04 μg/L). Moreover, the difference between the two treatment groups also showed significant variation (*p* < 0.001). But no differences were observed in the selenium content of liver, kidney, and muscle (Figures 1C–E), suggesting that the selenium might not accumulate in the organs.

To determine the selenium retention of hens after being fed DCH, we analyzed the selenium content in the egg (Figure 1F). We found that the egg selenium of hens treated with a 0.05 g/kg DCH diet (0.35 mg/kg) was significantly higher (*p* < 0.05) than the control group, but not in the 0.01 g/kg DCH group (0.27 mg/kg). These results suggested that the selenium *in vivo* was transported *via* blood and eventually accumulated in eggs consistent with other studies (28).

### DCH enhances the antioxidant activity and IgG level of hen

Selenium was noted for its antioxidative capability to regulate the oxidant balance and avoid diverse illnesses (19). To further understand the efficacy of selenium in DCH, we measured the SOD and CAT levels in serum. As shown in Figures 1G,H, the levels of CAT and SOD in serum were significantly increased in a dose-dependent manner after adding DCH for 30 days (*p* < 0.05), in line with serum Se content. Furthermore, we found that the CAT of the high-dose group increased from day 20 as well as the SOD of two treatment groups had a significant increase from day 10 after being fed the DCH (Supplementary Figures 1F,G). These data suggested that DCH could improve the antioxidant ability by enhancing the levels of the antioxidant enzyme. Consistent with the results of our work, selenium supplement enhanced the antioxidant capacity through upregulating CAT and SOD levels of laying hens in other studies (29–31).

Moreover, the moderate improvement of selenium intake was a benefit to adaptive immunity by increasing the levels of immunoglobulin and regulating the selenoprotein gene expression (17, 32). Then the IgG level was analyzed to evaluate the immune efficacy of DCH. We observed that the IgG level of hens was significantly increased in response to treatment with 0.05 g/kg DCH, reaching 99.13 μg/ml, but there was a decrease in the low-dose group (Figure 1I). Next, to clarify the significant reduction of the 0.01 g/kg DCH group, we compared the IgG levels of three groups on days 10 and 30, respectively. As shown in Supplementary Figure 3B, IgG levels were increased after feeding 0.01 and 0.05 g/kg DCH diet compared that no significant change in the control group (*p* > 0.05), suggesting that DCH probably improved the immunity of laying hens in 30 days.

### DCH feeding improves the laying performance

**Figure 2:**
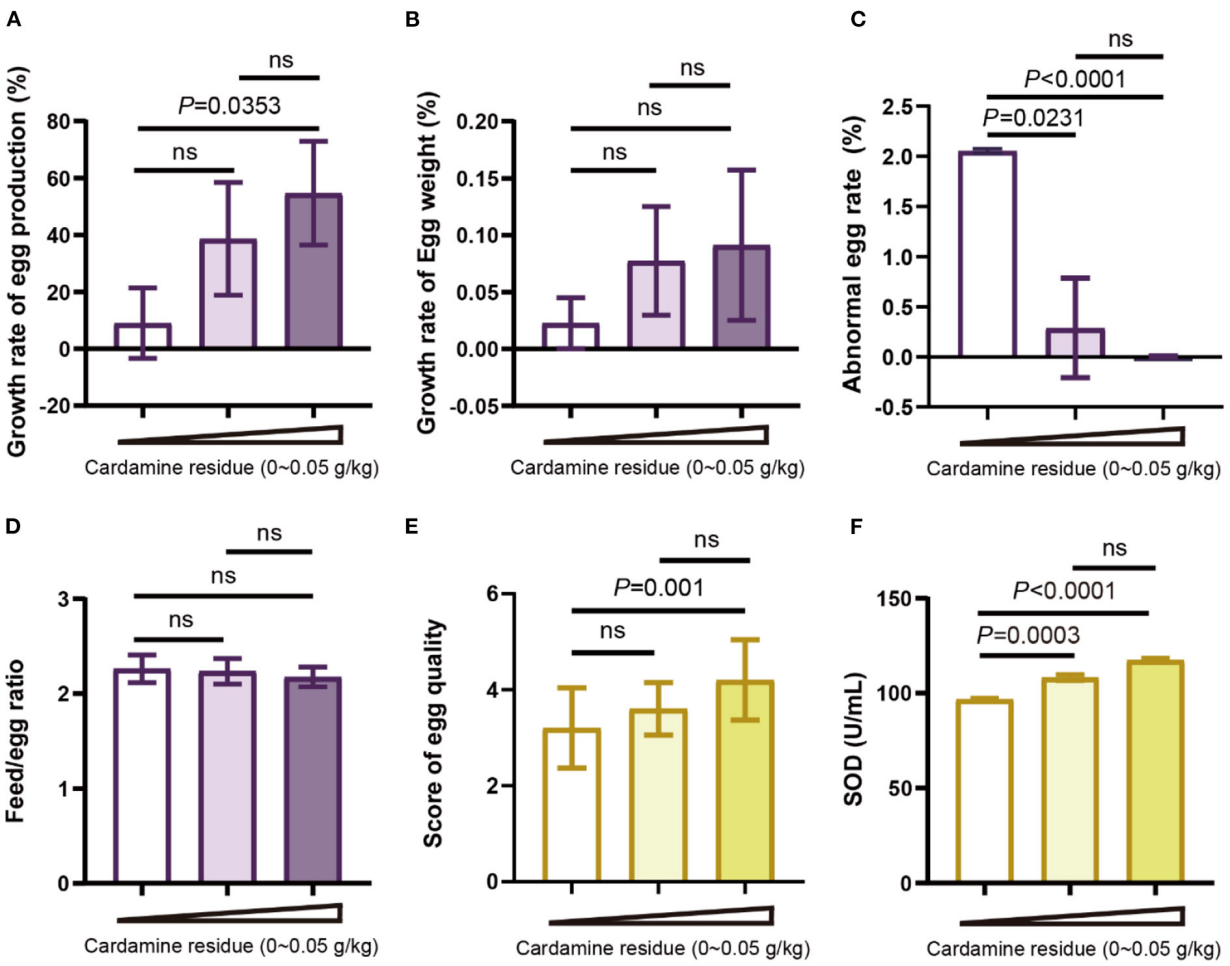
Effects of DCH on production performance and egg quality of laying hens. **(A)** Growth rate of egg production from day 0 to day 30. **(B)** Growth rate of egg weight from day 0 to day 30. **(C)** Abnormal egg rate of day 30. **(D)** The feed-egg ratio of each group after 30 days of experiment. **(E)** Egg quality scoring for each group. **(F)** SOD in eggs of each group.

To investigate the laying performance of hens after feeding DCH, we observed the growth rate of egg production and egg weight as well as the abnormal egg rate. We found that the growth rate of egg production in the high-dose group (54.68%) was significantly increased, whereas no difference in the low-dose group (38.65%) (Figure 2A). The improvement in egg production growth rate is shown from day 20 (Supplementary Figure 2A), indicating that DCH could accelerate the egg production of hens. Meanwhile, compared to the control group, the abnormal egg rate of low-dose and high-dose groups were significantly reduced by 0.29% and 0.003%, respectively (Figure 2C), suggesting that DCH promoted the success rate of laying hens. However, no significant difference was found in the growth rate of egg weight, which was increased dose-dependent after 30 days of DCH (Figure 2B).

Then we calculated the feed-to-egg ratio to evaluate the feed utilization. The result showed that there was no significant difference in the feed-to-egg ratio after feeding DCH for 30 days (Figure 2D). Taken together, these data suggested that treatments with 0.01 and 0.05 g/kg DCH diet could improve egg production performance, especially in the increase of egg production rate and downregulation of abnormal rate.

### DCH potentiates the egg quality

Next, we extended to examine the effect of DCH on egg quality. To evaluate the egg quality in a straightforward way, a standard of grading was formulated according to the egg shape index, yolk color, yolk ratio, eggshell thickness, and eggshell ratio shown in Table 1 and the total score was shown in Figure 2E. In line with the selenium content of the egg, the score of egg quality in the 0.05 g/kg DCH group was significantly higher than the control group (*p* < 0.01), whereas no significant difference was observed in the 0.01 g/kg DCH group. We found that indicators of egg quality including the egg shape index, yolk color, yolk ratio, eggshell thickness, and eggshell ratio all improved after DCH treatment (Supplementary Table 4), suggesting that DCH significantly potentiated the egg quality. Additionally, there may be a positive relationship between egg quality and egg selenium content. Moreover, we detected the SOD level in eggs and the result showed that the eggs in the 0.01 and 0.05 g/kg DCH groups had higher (*p* < 0.01) levels of SOD than those in the control group (Figure 2F), indicating that DCH not only increased the SOD content in serum but improved the SOD level in eggs.

## Discussion

Selenium is an essential trace element for the development of animals. There are varieties of selenium supplements used in the poultry industry to increase selenium *in vivo* and improve growth performance. In this study, we confirmed that the selenium *in vivo* was transported *via* blood and eventually accumulated in eggs rather than other organs including liver, kidney, and muscle. As shown in Supplementary Figure 1, the serum selenium content was significantly increased on day 10, indicating that selenium was absorbed in blood firstly *in vivo* and then transported to other tissues. Differenced with our results, the selenium was deposited in the liver and kidney of hens under 0.51 mg/kg selenite or Se-malt (33).

Increasing evidence shows that selenium supplement promote antioxidant capacity and animal immunity (19, 25, 34). The antioxidant effect of selenium is mainly reflected in the participation of selenium amino acids in the formation of protease (35). Diets supplemented with selenomethionine enhanced the SOD level and decreased oxidative reactions of laying hens (36). SOD catalyzed free radicals to produce oxygen and hydrogen peroxide *in vivo*, and then the hydrogen peroxide further decomposed into water and oxygen under CAT (37). In this work, dietary 0.01 and 0.05 g/kg DCH supplementation increased the contents of CAT and SOD in the serum of laying hens relative to the control group, indicating that DCH played an antioxidant effect by increasing the levels of antioxidant enzymes. This is consistent with previous reports that the addition of bacterial organic selenium or Se-yeast in the diet increased glutathione peroxidase (GSH-Px), CAT, and SOD activity in laying hens (38). Sun et al. used selenium-enriched earthworm powder as an additive to feed laying hens, observing the improvement of antioxidant activity and immune response (39). In addition, selenide glucose (SeGlu) supplementation improved the eliminative capacity of superoxide radicals in eggs (40). These studies showed that selenium supplement improved the antioxidant capacity of laying hens, which was almost consistent with the results of our study, but the effects of DCH on GSH-Px, a normal indicator of antioxidant ability need further tracking.

In fact, selenium supplements trigger the immune-stimulating effect, such as the activity of T cells, B cells, and natural killer cells, and increase the level of immunoglobulin (17, 41, 42). In addition, selenium can indirectly improve animal immunity through the antioxidant effect. For instance, during microorganisms invasion, a number of peroxide ions were released because of the activation and phagocytosis of immune cells, resulting in cell damage. Thus, the eliminative effect of peroxidase is particularly critical. The report showed that the selenium-enriched soybean peptides isolated from selenium-enriched soybean protein improved the immunomodulatory ability of cyclophosphamide-induced immunosuppressive mice (43). Several studies have shown that adding selenium supplements to the diet can improve the level of immunoglobulin *via* upregulation of SOD, CAT, and IgG in the serum of laying hens, indicating that selenium is a good immune regulator (39, 43, 44). This is consistent with our results that laying hens fed with 0.05 g/kg DCH showed a high level of SOD, CAT, and IgG, indicating that selenium-enriched DCH improved the immunity of hens as an economical feed additive. Though the 0.01 g/kg DCH group showed a lower level of IgG on day 30 compared to the control group, it was significantly increased after DCH feeding compare to day 10 of this low-dose group (Supplementary Figure 3B). The results of this and previous studies have shown the improvement of selenium supplements in the growth of laying hens. As for the specific mechanism of DCH increasing IgG level, it needs to be further explored.

**Figure 3:**
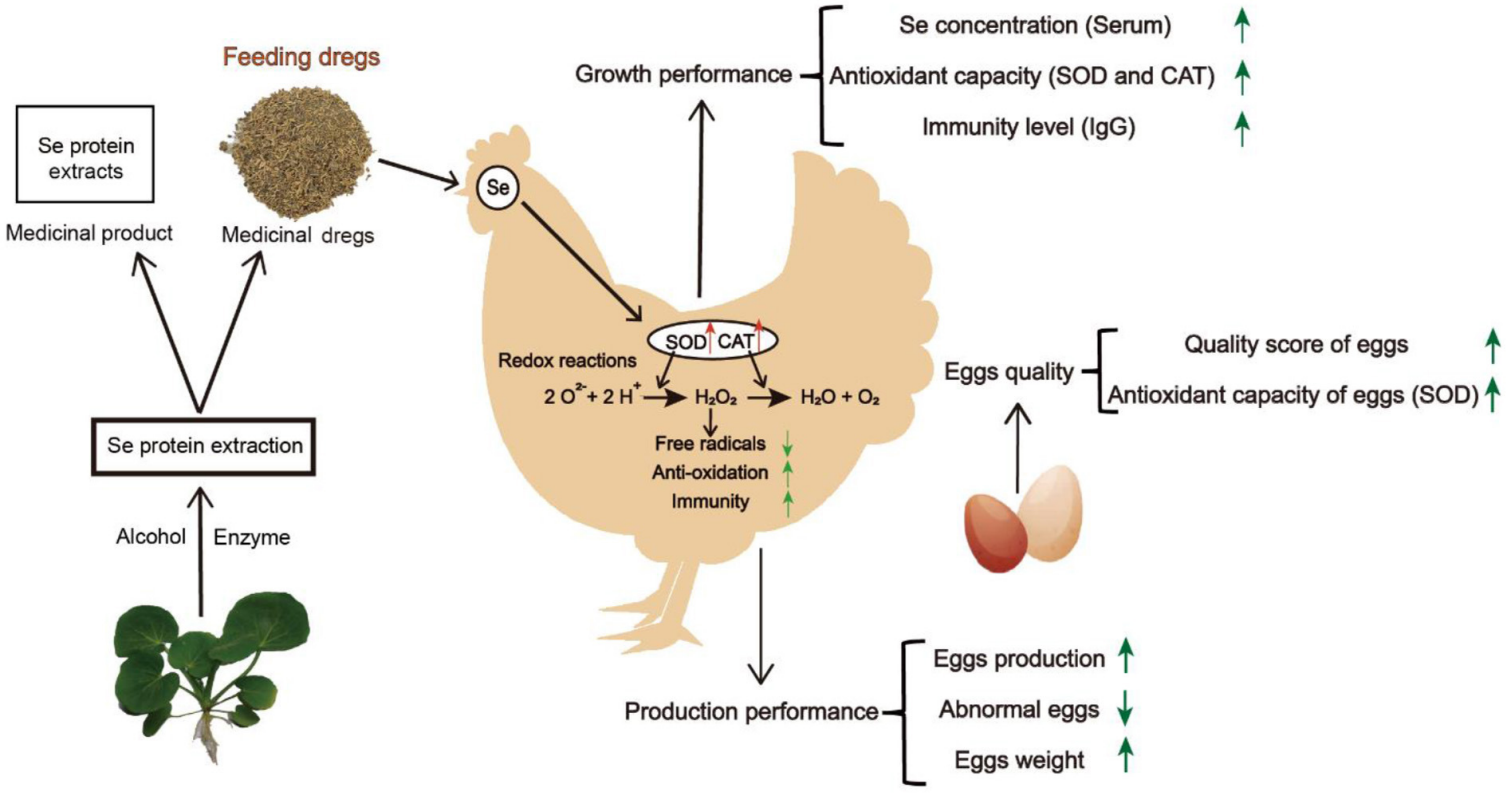
The DCH was added into diet as an additive to feed laying hens, which increased the content of serum selenium, improved the levels of SOD and CAT, accelerated the elimination of free radicals, improved the antioxidant capacity and immunity, and then enhanced the production performance and egg quality of laying hens.

As an economic animal, the most important thing is the production performance and egg quality of laying hens. Before the beginning of the test, we had detected the indicators of production performance and egg quality. As shown in Supplementary Tables 3, 4, the conditions of laying hens were no significant difference except for egg production, yolk color, yolk ratio, eggshell thickness, and eggshell ratio, which high-dose group was worse than the control. With the improvement of serum selenium, antioxidant enzyme level, and immunity of laying hens, our results showed that the production performance of laying hens, including egg-laying rate, abnormal egg rate, and egg weight, were improved, as well as the egg quality was also promoted. Therefore, in this study, the performance improvement of laying hens benefits from the application of DCH, realizing the reuse of waste and reducing the production cost (Figure 3). Consistent with other studies, adding selenium to the layer diet can improve product performance and egg quality including yolk ratio, shell ratio, egg shape, shell thickness, yolk color, and so on (45, 46), indicating that the DCH has the potential to use as a good feed additive to promote the growth and development of economic animals as well as improve economic benefits. This study of DCH provides an alternative strategy to improve egg quality and the development of novel feed additives.

## Conclusion

Dregs of *C. hupingshanensis* (DCH) is served as the feeding additive to improve the productivity of laying hens. Diets supplemented with 0.01 g/kg and 0.05 g/kg DCH increase the content of selenium in serum and raise the levels of CAT and SOD relative to the control group. The selenium provided by the 0.05 g/kg DCH group is deposited in eggs relative to the control group. The antioxidant capacity and enhanced immune function are associated with the additive of DCH. Diets supplemented with 0.01 and 0.05 g/kg DCH significantly improve the production performance and egg quality of hens relative to a normal diet. These results demonstrate the possibility of DCH supplementation in the growth of laying hens, realizing the secondary utilization of herbal dreg resources.

## Data availability statement

The raw data supporting the conclusions of this article will be made available by the authors, without undue reservation.

## Ethics statement

The animal study was reviewed and approved by Genentech Institutional Animal Care and Use Committee at the Beijing University of Agriculture. Written informed consent was obtained from the owners for the participation of their animals in this study.

## Author contributions

HD and XL conceived the projects. XL, HD, FY, and XY performed the research design. FY, XY, RL, DG, QD, and BL performed the experiments and performed data analysis. All authors have read and agreed to the published version of the manuscript.

## Funding

This work was supported by the Natural Science Foundation of Beijing, China (Grant Nos. 6212005 and 6224060), 2022 Research and Innovation Ability Improvement Plan for Young Teachers of Beijing University of Agriculture (Grant No. QJKC2022028), Beijing University of Agriculture Science and Technology Innovation Sparkling Support Plan (Grant No. BUA-HHXD2022010), and Unveiling the List Project of Beijing University of Agriculture (Grant No. 2021085).

## Conflict of interest

The authors declare that the research was conducted in the absence of any commercial or financial relationships that could be construed as a potential conflict of interest.

## Publisher's note

All claims expressed in this article are solely those of the authors and do not necessarily represent those of their affiliated organizations, or those of the publisher, the editors and the reviewers. Any product that may be evaluated in this article, or claim that may be made by its manufacturer, is not guaranteed or endorsed by the publisher.
