# Corrigendum: Dregs of *Cardamine hupingshanensis* as a feed additive to improve the egg quality

**DOI:** 10.3389/fnut.2023.1288837

**Published:** 2023-09-27

**Authors:** Feike Yu, Xiaohan Yu, Rongchen Liu, Dawei Guo, Qian Deng, Bingbing Liang, Xiaoye Liu, Hong Dong

**Affiliations:** ^1^Beijing Key Laboratory of Traditional Chinese Veterinary Medicine, Beijing University of Agriculture, Beijing, China; ^2^Beijing Traditional Chinese Veterinary Engineering Center, Beijing University of Agriculture, Beijing, China

**Keywords:** dregs of *Cardamine hupingshanensis*, feed additive, laying hens, production performance, egg equality

In the published article, there was an error. A figure panel was incorrectly cited. A correction has been made to **Results**, *DCH increases selenium in hen serum and egg*, paragraph two, second line.

This sentence previously stated:

“We analyzed the selenium content in the egg (Figure 1B).”

The corrected sentence appears below:

“We analyzed the selenium content in the egg (Figure 1F).”

In the published article, there was an error regarding the dosage unit for DCH. A correction has been made to **Conclusion**.

This sentence previously stated:

“Diets supplemented with 0.01 mg/kg and 0.05 mg/kg DCH increase the content of selenium in serum and raise the levels of CAT and SOD relative to the control group. The selenium provided by the 0.05 mg/kg DCH group is deposited in eggs relative to the control group. The antioxidant capacity and enhanced immune function are associated with the additive of DCH. Diets supplemented with 0.01 and 0.05 mg/kg DCH significantly improve the production performance and egg quality of hens relative to a normal diet.”

The corrected sentence appears below:

“Diets supplemented with 0.01 g/kg and 0.05 g/kg DCH increase the content of selenium in serum and raise the levels of CAT and SOD relative to the control group. The selenium provided by the 0.05 g/kg DCH group is deposited in eggs relative to the control group. The antioxidant capacity and enhanced immune function are associated with the additive of DCH. Diets supplemented with 0.01 and 0.05 g/kg DCH significantly improve the production performance and egg quality of hens relative to a normal diet.”

In the published article, there was an error. An author was inadvertently excluded from the **Author contributions** section.

A correction has been made to **Author contributions**.

This sentence previously stated:

“FY, XY, RL, DG, and BL performed the experiments and performed data analysis.”

The corrected sentence appears below:

“FY, XY, RL, DG, QD, and BL performed the experiments and performed data analysis.”

The authors apologize for these errors and state that this does not change the scientific conclusions of the article in any way. The original article has been updated.

