# Supplementary material for: Dregs of Cardamine hupingshanensis as a feed additive to improve the egg quality
Source: Front Nutr. 2022 Jul 28;9:915865. doi: 10.3389/fnut.2022.915865 (PMC9366334; doi:10.3389/fnut.2022.915865)
Supplement: Supplementary file 1 [file Data_Sheet_1.docx]

Supplementary Material

# Supplementary Tables

**Supplementary Table 1.** The groups of hens.

| **Group Name** | **DCH added in diets** |
| --- | --- |
| Control group | 0 g/kg |
| Low-dose *Cardamine* group | 0.01 g/kg |
| High-dose *Cardamine* group | 0.05 g/kg |

**Supplementary Table 2.** Composition and nutrient levels of the basal diet.

| **Composition (%)** | | **Nutrient levels (%)** | |
| --- | --- | --- | --- |
| Corn | 60 | Metabolic energy | 11.35 |
| Soybean meal | 25 | Crude protein | 15.66 |
| Soybean oil powder | 3 | Calcium* | 3.4 |
| Stone powder | 7 | Available phosphorus* | 0.43 |
| Premix | 5 | Selenium* | 0.045 |
|  |  | Methionine | 0.43 |
|  |  | Lysine | 0.96 |
| Total | 100 | Cystine | 0.31 |

*The premix provides vitamin A 150000 IU, vitamin D3 50000 IU, vitamin E 230 mg, vitamin K3 20 mg, vitamin B1 31 mg, vitamin B2 105 mg, vitamin B6 63 mg, vitamin B12 0.3 mg, niacin 460 mg, pantothenic acid 168 mg, folic acid 12.6 mg and phytase 6000 U per kilogram of diet.*

**Represents the measured value, and the rest are calculated values*

**Supplementary Table 3. Production performance of hens during added DCH.**

|  | Group | Day | | | |
| --- | --- | --- | --- | --- | --- |
|  |  | Before | 0-10 d | 11-20 d | 21-30 d |
| Egg Production | Control | 68.18±2.85^a^ | 76.59±2.0^a^ | 78.4±1.41^b^ | 74.1±5.97^b^ |
|  | Low-dose | 64.55±3.86^ac^ | 81.21±7.6^a^ | 92.44±3.95^a^ | 89.07±9.17^a^ |
|  | High-dose | 54.62±2.05^b^ | 77.42±10.65^a^ | 90.87±13.13^ac^ | 84.26±6.9^ac^ |
|  |  |  |  |  |  |
| Mean Egg Weight | Control | 57.61±1.08^a^ | 58.03±1.3^a^ | 58.02±1.0^c^ | 58.93±2.05^a^ |
|  | Low-dose | 56.24±1.64^a^ | 59.62±1.8^a^ | 60.03±0.6^a^ | 60.53±0.92^a^ |
|  | High-dose | 55.45±3.17^a^ | 60.54±3.4^a^ | 59.9±0.19^b^ | 60.37±0.4^a^ |
|  |  |  |  |  |  |
| Feed-egg Ratio | Control | 2.16±0.35^a^ | 2.21±0.31^a^ | 2.05±0.1^a^ | 2.16±0.14^a^ |
|  | Low-dose | 2.09±0.06^a^ | 2.24±0.08^a^ | 2.07±0.06^a^ | 2.04±0.14^a^ |
|  | High-dose | 2.08±0.19^a^ | 2.13±0.27^a^ | 2.04±0.06^a^ | 2.08±0.11^a^ |
|  |  |  |  |  |  |
| Abnormal egg ratio | Control | 1.19±1.19^a^ | 2.03±1.78^a^ | 1.72±0.77^a^ | 2.06±0.025^a^ |
|  | Low-dose | 1.51±1.3^a^ | 0.68±1.17^a^ | 0.69±0.68^b^ | 0.29±0.5^ac^ |
|  | High-dose | 1.24±0.84^a^ | 0.6±0.52^a^ | 0.82±0.94^ab^ | 0.03±0.05^b^ |
|  |  |  |  |  |  |

Values in a column with different superscripts are significantly different at P<0.05

**Supplementary Table 4. Egg quality of hens during added DCH.**

|  | Group | Day | | | |
| --- | --- | --- | --- | --- | --- |
|  |  | 0 d | 10 d | 20 d | 30 d |
| Egg shape index | Control | 1.25±0.02^a^ | 1.25±0.03^b^ | 1.23±0.02^b^ | 1.25±0.03^b^ |
|  | Low-dose | 1.24±0.03^a^ | 1.26±0.03^b^ | 1.27±0.04^b^ | 1.28±0.03^b^ |
|  | High-dose | 1.23±0.05^a^ | 1.31±0.01^a^ | 1.31±0.01^a^ | 1.32±0.02^a^ |
|  |  |  |  |  |  |
| Yolk color | Control | 8.86±0.19^a^ | 8.67±0.19^a^ | 8.72±0.11^c^ | 8.86±0.18^b^ |
|  | Low-dose | 8.36±0.14^b^ | 8.72±0.11^a^ | 8.97±0.22^b^ | 9.05±0.13^b^ |
|  | High-dose | 8.67±0.11^ac^ | 8.89±0.17^a^ | 9.5±0.15^a^ | 10.41±0.24^a^ |
|  |  |  |  |  |  |
| Yolk ratio | Control | 23.71±0.21^b^ | 23.2±0.06^c^ | 24.06±0.13^b^ | 24.1±0.17^c^ |
|  | Low-dose | 23.97±0.09^a^ | 25.17±0.13^b^ | 26.44±0.17^ac^ | 26.38±0.13^b^ |
|  | High-dose | 23.06±0.11^c^ | 26.12±0.13^a^ | 26.56±0.13^a^ | 26.58±0.12^a^ |
|  |  |  |  |  |  |
| Eggshell thickness | Control | 0.34±0.02^ab^ | 0.33±0.01^b^ | 0.34±0.02^b^ | 0.34±0.01^b^ |
|  | Low-dose | 0.36±0.01^a^ | 0.36±0.01^a^ | 0.38±0.03^ac^ | 0.39±0.01^ac^ |
|  | High-dose | 0.33±0.01^b^ | 0.36±0.02^a^ | 0.39±0.01^a^ | 0.4±0.01^a^ |
|  |  |  |  |  |  |
| Eggshell ratio | Control | 8.4±0.11^b^ | 8.27±0.11^c^ | 8.5±0.16^c^ | 8.54±0.06^c^ |
|  | Low-dose | 8.78±0.15^a^ | 8.95±0.07^b^ | 9.7±0.08^a^ | 9.67±0.14^a^ |
|  | High-dose | 8.29±0.08^b^ | 9.04±0.06^a^ | 9.4±0.12^b^ | 9.33±0.12^b^ |
|  |  |  |  |  |  |

Values in a column with different superscripts are significantly different at P<0.05

# Supplementary Figures


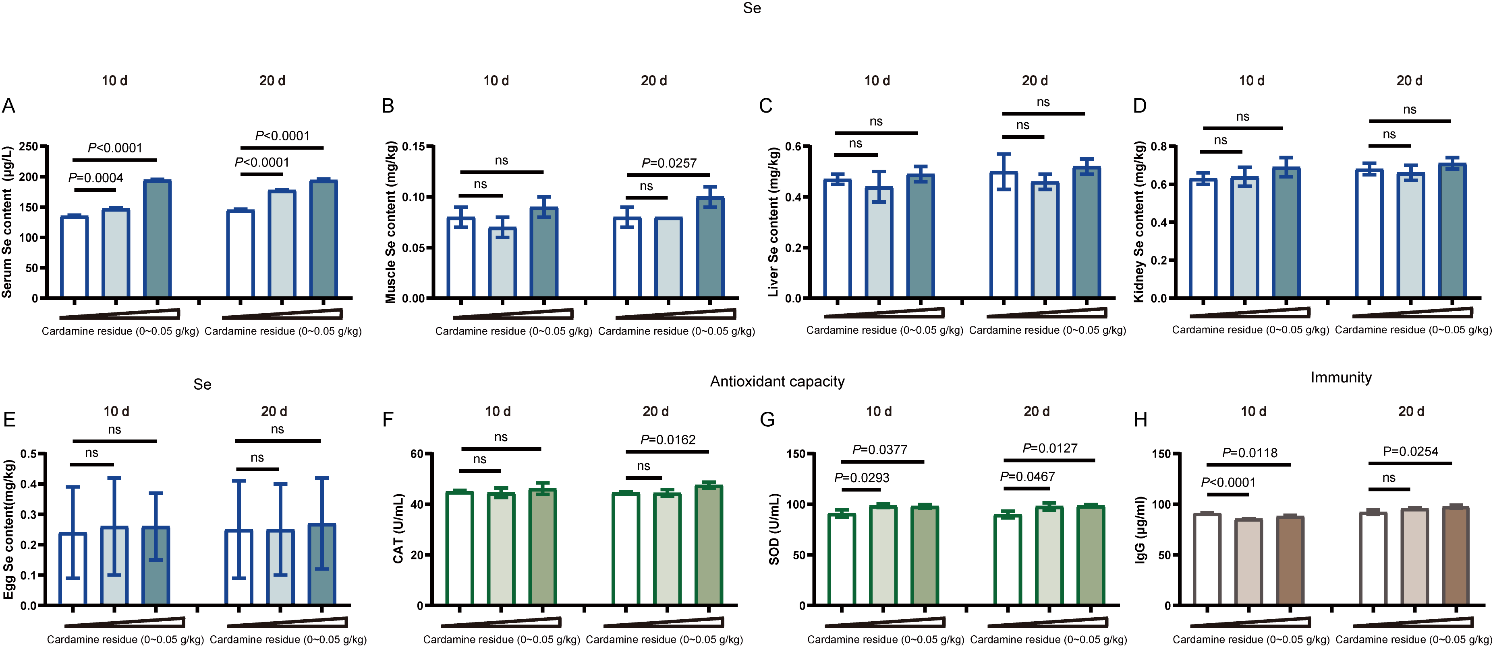


**Supplementary Figure 1. Effects of DCH on serum selenium, organs selenium, egg selenium, antioxidant enzyme levels and IgG level on day 10 and day 20.** (A) Selenium in serum of each group. (B) Selenium in muscle of each group. (C) Selenium in liver of each group. (D) Selenium in kidney of each group. (E) Selenium in eggs of each group. (F, G) The levels of antioxidative enzymes of SOD and CAT in serum of laying hens in each group. (H) IgG level in serum of laying hens in each group.


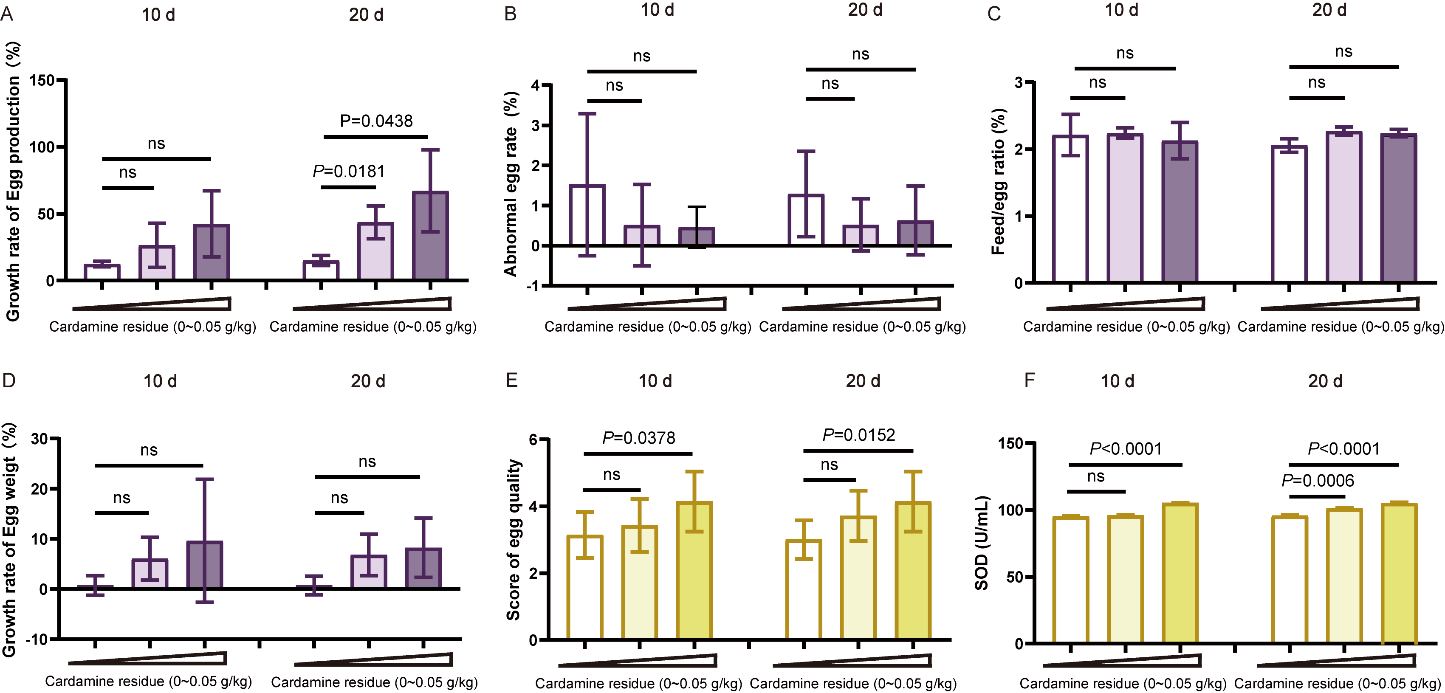


**Supplementary Figure 2. Effects of DCH on production performance and egg quality of laying hens** (A) Growth rate of egg production of first period (from day 0 to day 10) and second period (from day 11 to day 20). (B) Abnormal egg rate of first period and second period. (C) The feed-egg ratio of first period and second period. (D) Growth rate of egg weight of first period and second period. (E) Egg quality scoring for each group of first period and second period. (F) SOD in eggs of first period and second period.


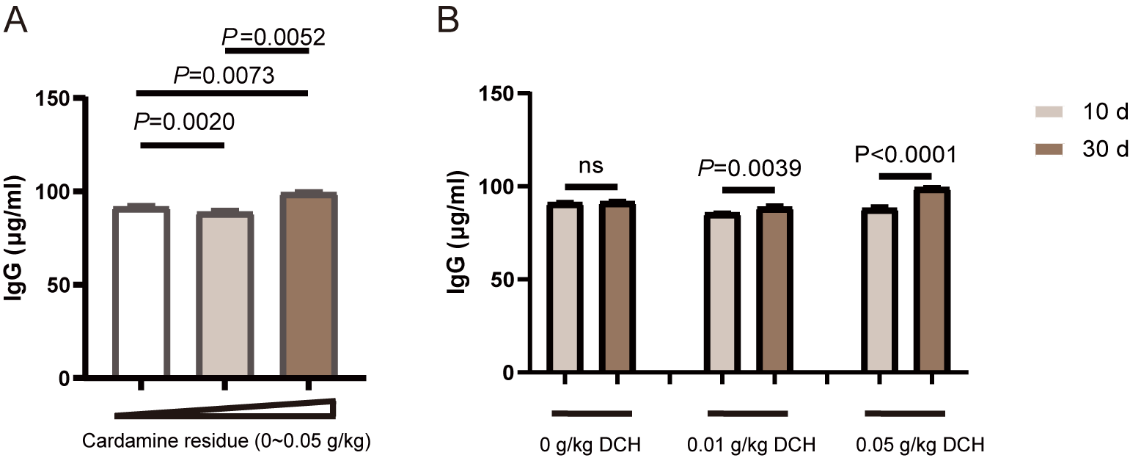


**Supplementary Figure 3. Effects of DCH on immunity of laying hens.** (A) IgG level on day 30 of three groups. (B) IgG level on day 10 and day 30 of three groups.
